# Pain-related health care costs for long-term care residents

**DOI:** 10.1186/s12877-021-02424-2

**Published:** 2021-10-14

**Authors:** Harminder Guliani, Thomas Hadjistavropoulos, Shan Jin, Lisa M. Lix

**Affiliations:** 1 Department of Economics, University of Regina, 3737 Wascana Pkwy, Regina, SK S4S 0A2 Canada; 2 Department of Psychology and Centre on Aging and Health, University of Regina, 3737 Wascana Pkwy, Regina, SK S4S 0A2 Canada; 3 Saskatchewan Health Quality Council, 241 – 111 Research Drive Saskatoon, Saskatoon, SK 7N 3R2 Canada; 4 Department of Community Health Sciences, University of Manitoba, S113-750 Bannatyne Ave, Winnipeg, MB R3E 0W3 Canada

**Keywords:** Direct health care cost, Nursing home, Clinically significant pain, Older adults, Canada

## Abstract

**Background:**

We tested for differences in direct health care costs among long-term care (LTC) residents age 65 and older with clinically significant pain (CSP) and with no pain or non-daily mild pain (NP/NDMP). We are not aware of any other large scale investigation that examined the cost of pain in LTC environments.

**Methods:**

Population-based administrative health data from Saskatchewan, Canada for 2004 to 2015 were used to compare direct health care costs for CSP and NP/NDMP groups up to one year after admission to LTC. Total accumulated costs for hospitalization, physician services, LTC, and prescription drugs were calculated in 2015 Canadian dollars. Group differences were tested using generalized linear models with generalized estimating equations.

**Results:**

Amongst 24,870 LTC residents, 8289 (33.3%) were censored due to death or discharge in the 365-day study observation period. Of the 16,581 (66.7%) observed residents, 5683 (34.3%) had CSP at admission. Residents (66.3% female) had a mean age of 85 years (SD = 7.4). The mean annual total direct health care cost per resident was higher among the CSP group (CAD $8063) than the NP/NDMP group (CAD $6455). This difference was found even after including LTC costs, and for each cost component (i.e., CSP residents had higher hospitalization, physician, and prescription drug costs). Similar results were obtained after controlling for demographics, comorbidities, physical and cognitive impairment, prior health care costs, and facility characteristics.

**Conclusion:**

The higher costs incurred by CSP residents compared to NP/NDMP residents are likely underestimated because pain problems are often missed in residents with dementia, who comprise a large portion of the LTC population. Improved pain care can reduce such costs and improve quality of life.

**Supplementary Information:**

The online version contains supplementary material available at 10.1186/s12877-021-02424-2.

## Introduction

Prevalence estimates for pain in residents of long-term care (LTC) facilities range from 40 to 85% depending on study methods and the population under investigation [1]. Despite this high prevalence, pain is often undertreated among LTC residents, many of whom have cognitive impairments [1, 2]. People with cognitive impairments, for example, are less likely to receive analgesics than their cognitively intact counterparts [3, 4]. This occurs, at least in part, because of limitations in ability to communicate subjective states such as pain due to cognitive decline [5] which affects a large portion of LTC residents. There is a paucity of research on the cost implications of pain in LTC environments.

Although pain care in LTC can be improved with increased assessment focusing on well validated observational methods that focus on pain behaviours [6, 7], resource constraints are often cited as barriers to improving care and to conducting frequent pain assessments [8]. It is difficult to fully address resource constraints without having a full understanding of health care costs incurred for residents with clinically significant pain (CSP) as compared to those with no pain or with non-daily mild pain (NP/NDMP). If those who suffer from CSP incurred higher costs, the policy change for improved pain care would seem more feasible.

Researchers from several parts of the world have examined the cost of pain. In Canada, the direct care costs of pain have been estimated at CAD $6 billion per year [9, 10]. Those on waiting lists for access to pain clinics have been estimated to spend a median of CAD $17,544 for various expenses related to funding private treatments and lost productivity [11]. Using a large, population-based sample of adolescents and adults, Hogan et al. [12] found that the annual incremental per-person cost to manage chronic pain in Ontario, Canada was CAD $1742, which is 51% higher than health care costs for patients without chronic pain. These researchers also reported that health care costs were highest in patients reporting more severe pain and more activity limitations [12]. Similarly, Lalonde et al., [13] estimated the economic burden of chronic noncancer pain in primary care patients in the province of Quebec, Canada. They found that the total direct health care costs averaged CAD $ 9565 per patient per year and varied positively with the level of pain disability. The mean adjusted total direct health care costs averaged CAD $7374, CAD $10,524, and CAD $9546 for patients with mild, moderate, and severe pain disability, respectively [13].

In the United States, the incremental health care costs due to pain have been estimated as ranging between US $261 billion and $300 billion annually [14]. In their study, Gaskin and Richard [14] indicated that their cost estimates represent underestimates of the actual cost of pain in the United States because they excluded certain populations including residents of LTC facilities. Other studies from Europe and Australia also suggest increasing health care costs with an increase in pain severity [15–17]. Collectively, these studies indicate that the economic burden of pain is substantial. While much has been written on the economic cost of pain among the general population in Canada and elsewhere, very little is known about the cost of pain in LTC residents.

In a recent longitudinal investigation examining the health care utilization of Canadian LTC residents as a function of pain status, Guliani et al. [18] concluded that those with CSP (as compared to residents who had NP/NDMP) had a greater risk of hospitalization, specialist physician visit, follow-up general practitioner visit, and use of various medications falling under at least three classes after controlling for comorbidities and prior health care utilization. Although health care utilization may be correlated with cost, it is not equivalent to cost. For example, a patient may have five physician visits, but these visits may cost considerably less than one 24-h hospital stay.

This study was, therefore, aimed to extend the findings of Guliani et al. [18] by describing the health care costs of LTC residents with CSP (defined as daily pain or moderate to severe non-daily pain) and residents with NP/NDMP. We hypothesized that LTC residents with CSP would have higher average cost compared to residents with NP/NDMP. An understanding of differences in costs between these groups will help health policymakers design efficient strategies to reduce and manage pain in LTC facilities.

## Methods

### Study design and cohort

**Fig. 1:**
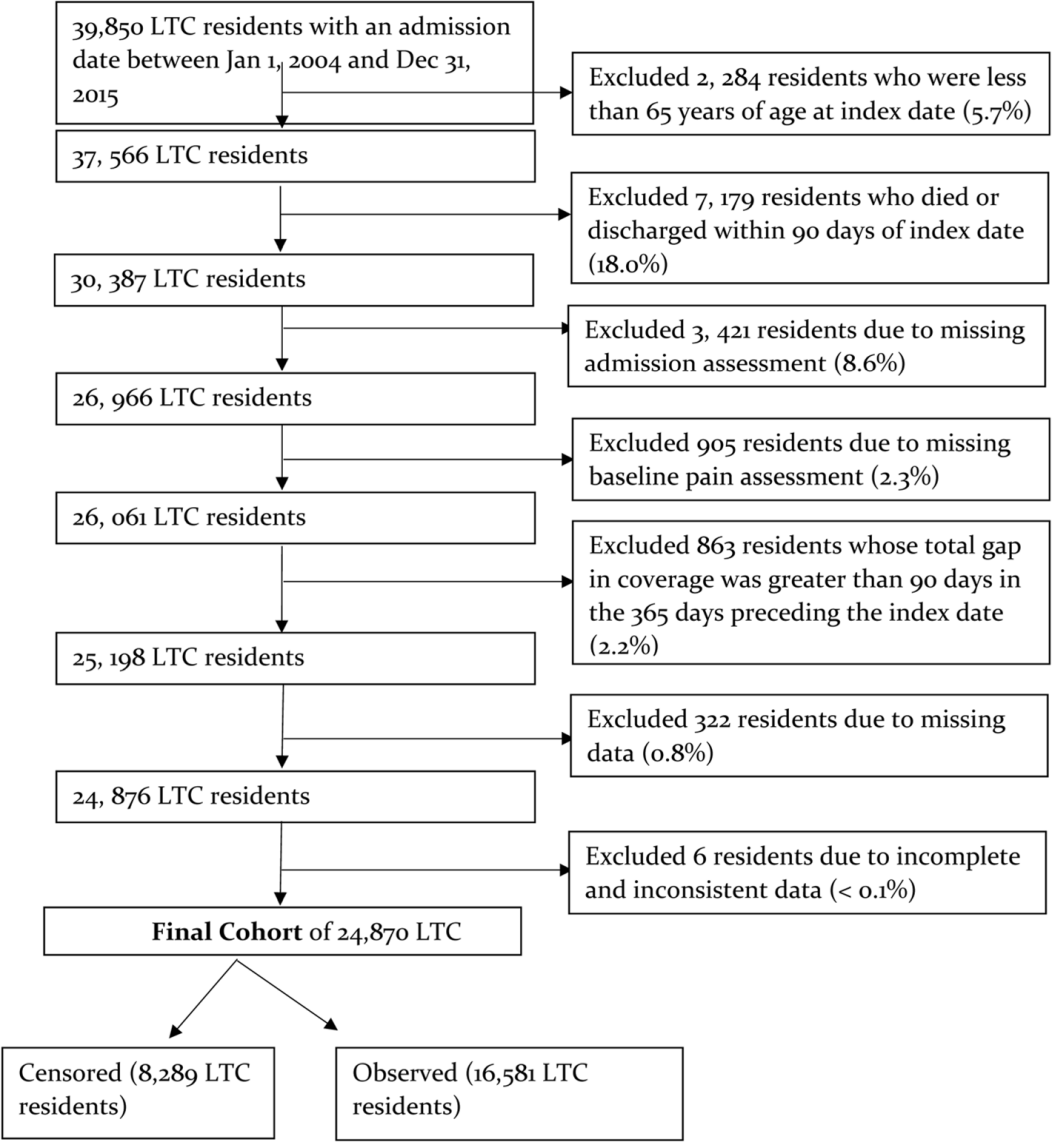
Cohort selection flow chart [18]

We used a one-year longitudinal cohort design. Our cohort included LTC residents with CSP and NP/NDMP. Specifically, the study cohort included all Saskatchewan LTC residents age 65 years and older, who were admitted to an LTC facility between January 1, 2004, and December 31, 2015, and had a minimum of 365 days of continuous health care coverage before their LTC admission date (i.e., index date). Residents from 165 LTC facilities, who were alive for at least 90 days after admission to LTC, were included. We followed cohort members from the date of LTC admission to 365 days after the index date, or until they were censored due to death or discharge from LTC, whichever came first. In total, there were 7179 cohort members excluded due to death or discharge within 90 days of admission, out of which 49% had pain. The excluded cohort members were comparable in baseline characteristics (e.g., 62% female with a mean age of 85 and a mean CPS score of 2) to included residents. Additionally, residents were excluded if they had no baseline pain assessment, had no LTC admission assessment, were less than 65 years of age at index date, or had incomplete or inconsistent data. The details of the cohort inclusion and exclusion criteria are reported in Fig. 1.

### Data sources

We used population-based administrative health data to track the cost of publicly funded health care services. These data have been described by Guliani et al. [18] and are available from the Saskatchewan Ministry of Health and eHealth Saskatchewan via the Saskatchewan Health Quality Council. With a population of approximately 1.1 million, [19] the Canadian province of Saskatchewan provides medically necessary care for all eligible residents, including outpatient physician visits, inpatient and outpatient hospital services, home care, LTC, and out-of-hospital prescription drugs.

Study data were from the Resident Assessment Instrument-Minimum Data Set (RAI-MDS) [20, 21], which is a clinical assessment tool for LTC residents, Person Health Registry System (PHRS), Discharge Abstract Database (DAD), prescription drug file, physician claims database, and the provincial Institutional Supportive Care Home (ISCH) and Special Care Home System (SCHS) database. Residents’ records from these databases were linked using unique personal health numbers and de-identified by replacing the personal health number with an unrelated study number. These Saskatchewan administrative health data cover nearly 99% of the province’s residents. The data exclude those who receive federal government health benefits including federal prison inmates, members of the armed forces, and the Royal Canadian Mounted Police.

The RAI-MDS captures clinical information about care, functioning, and health status of LTC residents. This information is collected on admission, quarterly, and whenever there is a major change in a resident’s health status [20, 21]. The PHRS provides residents’ health insurance coverage information, in addition to location of residence, and demographics.

The DAD provides information on all inpatient hospitalizations and day surgeries for patients of acute care facilities. Hospital abstracts are completed when a patient is discharged from an acute care facility, and diagnoses are recorded using the International Classification of Diseases, 10th revision, Canadian enhancement codes (ICD-10-CA) [22]. The physician claims database contains information on type and date of services, and a fee code that is used to determine the amount paid to the physician for each visit. A single diagnosis is recorded on each claim using a three-digit ICD-9 (i.e., 9th revision of ICD) code [23]. The adjudicated prescription drug database captures information on outpatient medications dispensed to people with provincial prescription coverage from September 1975 onward. However, prescription drug data were not consistently captured for LTC residents until 1996 because some LTC facilities received global funding for prescription drugs. That practice was discontinued by 1996. The prescription drug database excludes individuals who receive federal prescription coverage such as the military, federal police, federal prisoners, and Indigenous persons. Each available record includes the date of dispensation and a Health Canada drug identification number (DIN). The ISCH and SCHS database contains information on LTC facilities and length of stay for LTC residents. The ISCH database was decommissioned in 2013 and residents were migrated to the new Special Care Home System (SCHS) database in September 2013.

These databases have been used extensively for health services research in Saskatchewan [18, 24]. Other researchers have found Saskatchewan’s administrative health data to be reliable and complete [24, 25]. The study was approved by the Research Ethics Board, University of Regina. A standing data sharing agreement between the Saskatchewan Health Quality Council, the Ministry of Health, and eHealth Saskatchewan provided for access to the study data.

### Study variables

#### Health care costs

Direct health care costs, expressed in Canadian dollars, were estimated for each LTC resident from the date of LTC admission until death, discharge, or the end of the 365-day study observation period. The total accumulated cost for each LTC resident was the sum of costs for hospitalization (both inpatient and outpatient [i.e., day surgery]), general and specialist physician services, LTC residence, and dispensed prescription drugs. All costs were adjusted for inflation using 2015 dollars and the Saskatchewan health care component of the Consumer Price Index (CPI) from the Canadian Socio-Economic Information Management System (CANSIM [26];). Costs were also cumulated for the 365-day period prior to LTC admission and were used to control for confounding in the statistical models.

We estimated the total cost using both micro-costing and macro-costing approaches [27, 28]. The micro-costing approach, which combines appropriate unit costs with person-level utilization data (quantity x unit cost), was adopted to calculate the cost of physician services and prescription drugs. The macro-costing approach, commonly referred to as standard (or average) cost per service provided, was adopted to calculate the cost of inpatient hospitalizations, outpatient hospitalizations, and LTC stays.

The total hospital cost for each LTC resident was calculated by multiplying the cost of a standard hospital stay with its Resource Intensity Weight (RIW). The cost of a standard hospital stay for day surgery or inpatient hospital stay is the province-specific average direct cost per weighted case, which measures the province’s average total cost of treating an average acute inpatient for a given fiscal year [29]. We used the same values for day surgeries and inpatient stays, which may have resulted in some overestimation of costs. Nonetheless, this should not be a major concern since day surgeries accounted for only 9% of the total number of hospitalization records in our data. The RIW measures the intensity of resource utilization associated with different diagnostic procedures, surgical procedures, and demographic characteristics of an individual [30]. RIWs are assigned according to Case-Mix Groups (CMG), a patient classification algorithm developed by the Canadian Institute for Health Information, a national not-for-profit organization that provides information on the Canadian health care system [31]. CMGs classify patients into homogeneous groups based on similar clinical and resource-utilization characteristics. It should be noted that our hospital cost estimates may underestimate the total cost of hospitalization as it excludes hospital operational and building capital costs (e.g., hospital administration, utilities, or other capital-related costs) as well as visits to ambulatory clinics such as emergency departments.

The cost of physician services for each LTC resident was calculated by summing the fees for all physician visits during the study period. These costs were calculated separately for general practitioners and specialist physicians. In the case of non-fee-for-service physician payments, shadow billing claims were used to compute physician costs using the equivalent fee-for-service value. Some non-fee-for-service physicians may not submit shadow-billed claims. However, existing research supports the validity and completeness of the physician claims data for capturing the vast majority of physician services [25, 32].

The prescription drug cost for each LTC resident was the total cost for all dispensed outpatient prescriptions; this included drug materials, dispensing fee, and markup. Costs for drugs used in acute care facilities are captured in the total hospital cost.

The cost of LTC services for each LTC resident was determined by multiplying the annual per-diem cost by the number of days a member of the study cohort was an LTC resident. The annual per diem cost was calculated by dividing the same value of annual LTC provincial expenditures for all facilities by the total number of resident days. This information was obtained from the Ministry of Health Community Care Branch.

#### Covariates

The primary covariate of interest was pain group, which had two categories: CSP and NP/NDMP. This measure was derived from RAI-MDS assessment records, which captures information on pain frequency (i.e., no pain, pain less than daily, daily pain) and its intensity (i.e., mild pain, moderate pain, pain is horrible or excruciating) at the index date and quarterly thereafter. The RAI-MDS pain scale has been widely used for Canadian LTC residents [33] and its validity has been established [34]. Pain frequency and severity, stratified by sex is available in the Additional file accompanying this article. Similar to Guliani et al. [18], LTC residents with daily pain or less than daily pain that was of moderate or severe intensity were classified as having CSP. LTC residents with no pain or less than daily pain of mild intensity were classified as having NP/NDMP. Our approach to classification of patients in terms of their RAI-MDS pain status is consistent with quality indicator approaches used in Canada [35].

We also included the RAI-MDS Cognitive Impairment Scale (CPS [36]), Activities of Daily Living (ADL [36]), and Changes in Health, End-Stage Disease and Signs and Symptoms (CHESS [36]) as covariates in our regression analysis; these covariates measure residents’ physical and cognitive impairment. The CPS evaluates the level of cognitive impairment affecting a patient such as memory, decision-making ability, communication skills and eating impairments, and is scored on a seven-point scale with a minimum value of zero and a maximum value of six. Higher scores indicate more severe cognitive impairment [36]. We used the ADL long-form scale to measure residents’ self-sufficiency. The scale is composed of seven ADL items; total scale scores range from zero to 28, with higher scores indicating less independence [36, 37]. In our study, we categorized the ADL scores as 0–7, 8–14, 15–21 and 22–28. The CHESS scale is a measure of frailty and health stability designed to identify residents at risk of serious decline and takes on values from zero (no instability) to five (high instability). We have combined scores from three to five due to a small number of observations. A high CHESS score is shown to be a predictive of mortality in the LTC population [38]. Further details on these measures are provided by the Canadian Institute of Health Information [36].

Other covariates included residents’ demographic characteristics at the index date (age, sex, rural/urban residence), comorbidities, prior direct health care costs for the 365-days before the index date, and LTC facility characteristics. Age was categorized as 65–74, 75–79, 80–84, 85–89, 90–94, and 95+ years. A resident’s address from the PHRS at index date was used to classify the resident’s location as rural or urban. An address from one of the two health regions containing major urban centers was classified as urban, while an address from one of the remaining 11 health regions was classified as rural.

The Charlson comorbidity index (CCI [39];) score was calculated for the 365-day period prior to the index date. The index is based on ICD–9 and ICD–10-CA diagnosis codes captured in hospital and physician data [40, 41]. CCI scores were classified into four groups:, 0 (no comorbidities), 1, 2, and 3 or more comorbidities.

The natural log of total direct health care cost in the 365-day period prior to the index date was included as a covariate. The total health care cost was the sum of hospitalization, general practitioner visits, specialist visits, and prescription drug cost. These costs were adjusted for inflation using 2015 dollars and CPI for Saskatchewan health care component of CANSIM.

The Saskatchewan ISCH database classifies LTC facilities as integrated, special care homes, and hospital-based special care homes. Integrated LTC facilities provide the services of both an acute care facility and a special care home [42]. Special care homes are publicly funded and provide LTC services to qualified residents who require care and supervision that cannot be provided in their own homes [42]. Hospital-based special care homes serve individuals who are still in a hospital but do not require acute care services. The facility type variable allows us to capture any unknown and unmeasured variations in the services that facilities provide.

#### Statistical analysis

Frequencies, percentages, means, standard deviations (SD), and the interquartile range (IQR) were used to describe the cost data. Descriptive statistics were stratified by pain group at baseline (i.e., index date).

Generalized linear models (GLMs) with generalized estimating equations (GEEs) were used to test for differences in the marginal total direct health care costs between the CSP and NP/NDMP groups before and after adjusting for other covariates (i.e., unadjusted and adjusted models). GLM models are widely used for modelling health care costs [43, 44]. GLM with GEEs allowed us to account for the repeated measurement of costs within patients over time and to make robust inferences by implicitly considering the correlation structure of the data. The assumption of a normal distribution for the outcome, which underlies ordinary least-squares regression, is violated for cost data because it has a skewed distribution. We initially analyzed the data using GLMs with gamma and inverse Gaussian distributions and used a likelihood-based goodness-of-fit statistic to assess which distribution provided the best fit for the data. Based on the assessment of model fit, we selected a gamma distribution and an identity link function.

Pain group was defined as a time-varying covariate; it was updated each time a resident’s pain status changed based on information recorded during the RAI-MDS assessments. We used a time-varying covariate because pain status is unlikely to remain constant over time.

We first fit an unadjusted model to the cost data; it contained pain group, time, and the pain group x time two-way interaction. The two-way interaction enabled us to test whether the association of pain group with cost varied over time. We then fit a fully adjusted model that incorporated all patient and facility characteristics in addition to pain group, time, and the pain group x time two-way interaction.

Two adjusted and unadjusted models were fit to the data: In Model 1, the outcome variable was total direct health care cost including the LTC component. In Model 2 the outcome variable was total direct health care cost excluding the LTC component. By removing the LTC facility costs, we were able to analyze the impact of pain on the remaining health care services cost.

An exchangeable correlation structure, which assumes constant correlation in successive periods for the repeated measurements was used. The model offset was the natural log of the number of person-days of follow up (the number of days in the LTC) which accounts for differences in exposure time (i.e., due to censoring). GLM model fit was evaluated using the ratio of scaled deviance to the model degrees of freedom without considering correlation; a value close to one indicates a well-fitting model. A penalized quasi-likelihood fit statistic was used to evaluate fit when the correlation structure was taken into account. Regression coefficient estimates and 95% confidence intervals (CIs) are reported. SAS v.9.4 [45] was used to perform both the descriptive and inferential analyses.

## Results

### Cohort characteristics

Out of a total of 39,850 Saskatchewan LTC residents admitted between January 1, 2004 and December 31, 2015, almost two thirds (62.4%, *N* = 24,870) met the study inclusion criteria (Fig. 1). Of the 24,870 LTC residents, 8289 (33.3%) were censored due to death or discharge in the 365-day study observation period. Table 1 summarizes the study cohort characteristics for the residents stratified by pain group membership at the index date. A total of 16,581 residents were observed from the admission to the end of 365-day period, of which about one third had CSP at the index date (34.3%, *n* = 5683) and about the same number of residents had CSP at the end of the study observation period (33.3%, *n* = 5523). Among those who had CSP at the baseline (*n* = 5683), 66.4% (*n* = 3773) of them also reported CSP at the end of the study observation period Residents were mainly women (66.3%, *n* = 10,896), residing in rural areas (63.0%, *n* = 10,443), with a mean age of 85.0 years (SD = 7.4). CSP residents were more likely to be female (71.2%), had a higher direct health care costs one year prior to the study index date (mean = $38,815; SD = 42,868), and a CCI score of 3 or more (26.9%). At the time of admission to LTC, 14.6% of residents with CSP had moderate to very severe cognitive impairment (score of 4+), 10.1% had a CHESS scale of 3 to 5 and 14.5% had minimum level of functioning (ADL score of 22 to 28).

**Table 1:** Cohort characteristics stratified by clinically significant pain (CSP) and no pain or non-daily mild pain (NP/NDMP) at LTC admission

	CSP^a^ (*n* = 5683)	NP/NDMP^a^ (*n* = 10,898)	*p*-value
**Age in years: Mean (SD)**	85 (7.4)	85 (7.3)	0.205
**Age groups (years)**			
65–74	574 (10.1)	1089 (10.0)	0.835
75–79	690 (12.1)	1413 (13)	0.157
80–84	1214 (21.4)	2411 (22.1)	0.320
85–89	1550 (27.3)	2897 (26.6)	0.414
90–94	1208 (21.3)	2234 (20.5)	0.310
95+	447 (7.9)	854 (7.8)	0.950
**Sex**			
Females	4047 (71.2)	6939 (63.7)	<0.0001
Males	1636 (28.8)	3959 (36.3)	<0.0001
**Location of residence**			
Urban	2111 (37.2)	4027 (36.9)	0.845
Rural	3572 (62.9)	6871 (63.1)	0.881
**Charlson comorbidity index score**			
0	1551 (27.3)	3443 (31.6)	<0.0001
1	1576 (27.7)	3090 (28.5)	0.474
2	1027 (18.01)	1929 (17.7)	0.591
3+	1529 (26.9)	2436 (22.3)	<0.001
**Prior health care cost ($):**			
**Mean (SD)**	38,815 (42,868)	32,668 (42,826)	<0.001
**Median**	25,989	18,798	
**Cognitive Performance Scale (CPS)**			
Intact (score 0)	836 (14.7)	1066 (9.8)	< 0.0001
Borderline intact (score 1)	976 (17.2)	1343 (12.3)	< 0.0001
Mild impairment (score 2)	1004 (17.7)	1879 (17.2)	0.533
Moderate (score 3)	1605 (28.2)	3718 (34.1)	< 0.0001
Moderate/Severe impairment (score 4)	216 (3.8)	656 (6.0)	< 0.0001
Severe impairment (score 5)	517 (9.1)	1349 (12.4)	< 0.0001
Very severe impairment (score 6)	99 (1.7)	173 (1.6)	0.416
Missing	430 (7.6)	714 (6.6)	0.018
**CHESS Scale**			
0	1996 (35.1)	5115 (46.9)	< 0.0001
1	1608 (28.3)	2707 (24.8)	< 0.0001
2	1073 (18.9)	1666 (15.3)	< 0.0001
3–5	573 (10.1)	676 (6.2)	<0.001
Missing	433 (7.6)	734 (6.7)	0.042
**ADL: Mean (SD)**	12.19 (8.0)	9.99 (7.6)	0.280
**ADL categories**			
0–7	1736 (30.6)	4562 (41.9)	< 0.0001
8–14	1425 (25.1)	2767 (25.4)	0.702
15–21	1267 (22.3)	1866 (17.1)	< 0.0001
22–28	825 (14.5)	989 (9.1)	<0.0001
Missing	430 (7.8)	714 (6.6)	0.018
**Long term care facility type**			
Integrated	5121 (90.1)	9918 (91.0)	0.565
Special Care Home	266 (4.7)	459 (4.2)	0.171
Hospital/Special Care Home	296 (5.2)	521 (4.8)	0.239

Note: CHESS = Changes in Health, End-stage Disease and Symptoms and Signs; ADL-Activities of Daily Living.

^a^Frequencies (%) are reported unless otherwise noted

### Health care costs

Table 2 summarizes the annual direct health care costs stratified by pain group. The average cost per resident was calculated separately for CSP and NP/NDMP groups by dividing the total cost by the total number of observed residents in each group. It should be noted that the death rate was similar in both groups during the 365-day study observation period. Nearly 23% of the residents with CSP died during the study observation period versus 20% of residents with NP/NDMP.

**Table 2:** Mean (standard deviation) and interquartile range (IQR) of annual direct health care costs for long-term care residents with clinically significant pain (CSP) and no pain or non-daily mild pain (NP/NDMP) (CAD $)

Cost Component	CSP	NP/NDMP	Difference	*P*-value
	(*n* = 5683)	(*n* = 10,898)		
Hospital	3374 (9082) IQR: 1077	2645 (9330) IQR: 0^a^	729	<0.0001
Specialist Physicians	394 (1013) IQR: 311	319 (825) IQR: 245	74	<0.0001
General Practitioner Physicians	1040 (625) IQR: 920	990 (610) IQR: 959	49	<0.0001
Prescription Drug	3255 (2419) IQR: 2535	2500 (1891) IQR: 2085	755	<0.0001
Total, excluding LTC	8063 (10,493) IQR: 5632	6455 (10,257) IQR: 3943	1608	<0.0001
Total, including LTC	93,282 (11,118) IQR: 9188	91,406 (10,996) IQR: 8692	1876	< 0.0001

^a^The IQR for NP/NDMP group is zero for hospitalization as 78% of residents with NP/NDMP had zero hospitalization cost

Note: All costs are reported in 2015 constant Canadian dollars (CAD);

Residents with CSP reported higher health care costs than residents with NP/NDMP (Table 2). The mean annual total direct health care cost (excluding LTC costs) per resident was 24.9% higher for the CSP group (CAD $8063) compared to NP/NDMP group (CAD $6455). This unadjusted difference (CAD $1608) in cost by pain group was statistically significant (*p* <  0.0001). This pattern is consistent for each of the cost components, that is, LTC residents with CSP had higher costs for hospitalization, general and specialist physician visits, and prescription drugs. All of these cost type differences were statistically significant (p <  0.0001). Among the CSP group, hospitalization accounted for 41.9% of the total direct health care cost (CAD $3374/CAD $8063) followed by prescription drugs (40.4%), general practitioner visits (12.9%), and specialist visits (4.9%).

Among those with CSP at the time of admission, the top 10% of residents (in terms of costs incurred) accounted for 39.9% of the total direct health care cost (excluding LTC) and the bottom 10% (in terms of costs incurred) residents accounted for only 1.4% of the total costs. While the NP/NDMP group also had similar percentages, the mean annual direct cost per resident (for those whose costs fell at the top 10% of the distribution) was 19.0% higher in the CSP group (CAD $32,195 for CSP vs CAD $27,051 for NP/NDMP). Further examination of the data broken down by cost component shows that the top 10% of residents (in terms of costs incurred) with CSP accounted for 72.1% of the total hospitalization cost and for 67.1% of the total general physician costs. Similarly, the top 10% of residents (in terms of costs incurred) with NP/NDMP accounted for 78.0% of the total hospitalization costs followed by 68.9% of the total general physician costs. Nonetheless, pain group differences were noted for the residents who incurred the highest costs for both hospitalization (CAD $24,357 for CSP vs CAD $20,621 for NP/NDMP) and general physician costs (CAD $2642 for CSP vs CAD $ 2201) with those in the CSP group having the highest costs. A similar pattern was observed for specialist and prescription drug costs.

For those who were not discharged and did not die during the 365-day study period following LTC admission, the frequency of zero hospitalization and specialist cost was slightly higher among NP/NDMP residents (Table 3). While 78.3 and 49.4% of the NP/NDMP residents had no hospitalization or specialist cost, respectively, the corresponding percentages for CSP residents were 73.3 and 45.2% (Table 3). The overall distribution of total costs was bi-modal and skewed toward higher-cost residents, particularly for residents with CSP.

**Table 3:** Frequency of zero cost for long-term care residents during the 365-day study observation period following LTC admission with clinically significant pain (CSP) and no pain or non-daily mild pain (NP/NDMP)

	CSP (*n* = 5683)	NP/NDMP (*n* = 10,898)
Hospital	4163 (73.3%)	8413 (78.3%)
Specialist Physicians	2570 (45.2%)	5382 (49.4%)
General Physicians	98 (1.8%)	248 (2.3%)
Prescription Drugs	151 (2.7%)	333 (3.1%)

### Model results

Table 4 reports the unadjusted and fully-adjusted model estimates and 95% confidence intervals (CIs) for Model 1 and Model 2. Model 1 estimates are for cost data that include LTC costs, while Model 2 estimates are for cost data that exclude LTC costs. The ratio of the scaled deviance to the model degrees of freedom, using the gamma distribution, was close to one for both models (ratio was 1.0 in Model 1 with LTC costs and 1.2 in Model 2 without LTC costs) suggesting that both models fit the data reasonably well. The pain group x time interaction was statistically significant (*p* <  0.0001) in both models. The results from Model 2 (without LTC cost) were statistically significant (*p* <  0.0001) for most covariates.

**Table 4:** Regression model estimates and 95% confidence intervals (95% CIs), Gamma distribution

	Model 1		Model 2	
	Total cost including LTC cost		Total cost excluding LTC cost	
	Unadjusted	Adjusted	Unadjusted	Adjusted
	Estimate (95% CI)	Estimate (95% CI)	Estimate (95% CI)	Estimate (95% CI)
**Intercept**	**5.6494** (5.6352, 5.6637)	**5.6306** (5.5945, 5.6666)	**4.117** (4.2073, 4.2067)	**3.2628** (2.99, 3.5356)
**Clinically significant pain (**ref: No pain/Non-daily mild pain)	-0.0160 (-0.0341, 0.0022)	-0.0176 (-0.0356, 0.0003)	-0.1983 (-0.3152, 0.0813)	**-0.1538** (-0.2801, -0.0276)
**Time**	**-0.0004** (-0.0004, -0.0003)	**-0.0003** (-0.0003, -0.0003)	**-0.0034** (-0.0037, -0.0032)	**-0.0031** (-0.0033, -0.0027)
**Pain group x time**	**0.0001** (0.0001, 0.0002)	**0.0001** (0.0001, 0.0002)	**0.0014** (0.001, 0.0018)	**0.0011** (0.0007, 0.0015)
**Age** (ref: 65-74)				
75-79		-0.0112 (-0.0268, 0.0043)		-0.0584 (-0.1477, 0.0308)
80-84		**-0.0236** (-0.0379, -0.0093)		**-0.1268** (-0.2103, -0.0433)
85-89		**-0.0306** (-0.0446, -0.0116)		**-0.18** (-0.2625, -0.0976)
90-94		**-0.0362** (-0.0507, -0.0217)		**-0.2326** (-0.3299, -0.1423)
95+		**-0.0585** (-0.0734, -0.0436)		**-0.441** (-0.5466, -0.3416)
**Sex:** (ref: female) Male		0.0052 (0.0008, 0.0112)		**0.0898** (0.0452, 0.1344)
**Location of residence** (ref: Urban) Rural		-0.0043 (-0.0098, 0.0012)		**-0.0507** (-0.0953, 0.0061)
**Charlson comorbidity index score** (ref: 3+)				
0		**-0.0103** (-0.0155, -0.0051)		-0.0336 (-0.0785, 0.0114)
1		**-0.01** (-0.0146, -0.0054)		**-0.0683** (-0.1052, -0.0315)
2		-0.0005 (-0.0062, 0.0053)		-0.0103 (-0.0503, 0.0296)
**Prior health care cost**		**0.0047** (0.0019, 0.0074)		**0.0978** (0.0772, 0.1183)
**Cognitive Performance Scale (CPS) (ref: 0)**				
1		-0.0079 (-0.0207, 0.005)		-0.0814 (-0.1684, 0.0057)
2		**-0.026** (-0.0376, -0.0144)		**-0.1716** (-0.2557, -0.0876)
3		**-0.0273** (-0.0389, -0.0157)		**-0.233** (-0.3141,-0.1519)
4		**-0.0277** (-0.0429, -0.0125)		**-0.1946** (-0.3153, -0.0739)
5		**-0.0246** (-0.0375, -0.0118)		**-0.232** (-0.3253, -0.1388)
6		**-0.0388** (-0.0552, -0.0225)		**-0.3097** (-0.4444, -0.175)
**Activities of Daily Living (ADL): (ref: 0-7)**				
8-14		-0.0025 (-0.0105, 0.0055)		-0.0039 (-0.0641, 0.0562)
15-21		**-0.0116** (-0.0191, -0.004)		**-0.0923** (-0.1509, -0.0338)
22-28		**-0.0115** (-0.021, -0.0021)		**-0.1043** (-0.1733, -0.0353)
Changes in Health, End-Stage Disease and Signs and Symptoms **(CHESS) (ref: 0)**				
1		**0.0109** (0.0037, 0.0181)		**0.0806** (0.0261, 0.1352)
2		**0.0115** (0.0042, 0.0188)		**0.0882** (0.0333, 0.1431
3-5		**0.017** (0.0075, 0.0265)		**0.1249** (0.0559, 0.194)
**Long term care facility type** (ref: Integrated)				
Special care homes		**0.0687** (0.0378, 0.0996)		**0.4425** (0.2966, 0.6253)
Hospital/Special Care home		-0.0071 (-0.0182, 0.004)		-0.0324 (-0.1306, 0.0658)

*Note*: Bold values indicate statistically significant at α=0.05

The pain group x time interaction was positive and statistically significant in the unadjusted results (with and without including LTC costs) indicating that there were significant differences between the pain groups in cost changes over time. Even after adjusting for residents’ demographic characteristics, prior direct health care costs, and facility covariates, the pain group x time interaction was positive and statistically significant (*p* <  0.0001) in both models. The interaction estimate indicates that the change in cost over time depends on pain status. The average cost per day was higher in the CSP group than in the NP/NDMP group.

Estimates for the main effect of time revealed a decreasing trend (*p* <  0.0001) in both models. The coefficients on other covariates were in the expected direction. Women were more likely to have a higher cost than men. Costs increased with age group. The average health care cost per day was also higher among residents who had higher cost one year prior to the LTC admission and who had a greater level of comorbidity. Residents with higher health instability (as measured by CHESS score) at the baseline had a higher average cost per day than those with no health instability (CHESS score of 0). Results on CPS scale suggest that residents with severe cognitive impairment (CPS score of 6) had significantly lower average cost per day than those with intact cognition (CPS score of 0). Similarly, there is a significant negative association with ADL functioning and average cost per day. The predicted mean cost without including LTC cost for the CSP group was CAD $6099 and for the NP/NDMP group the predicted mean cost was CAD $5580.

## Discussion

To the best of our knowledge this is the first large scale investigation that estimated the cost of pain in an LTC environment by comparing direct health care costs for residents with CSP and those with NP/NDMP. As anticipated, the mean annual health care costs for Saskatchewan LTC residents with CSP on admission (excluding LTC costs) were 25% higher than those of NP/NDMP ($8062.53 vs. $6455). Specifically, the top 10% (in terms of health care cost) residents with CSP (measured at baseline) accounted for approximately 40% of total costs, with a mean annual direct cost per person of CAD $32,195. The results from the regression analysis further support our hypothesis that the average cost per day was higher in the CSP group than in the NP/NDMP group after controlling for various demographic characteristics, prior health care costs, measures of cognitive and physical functioning, and facility characteristics. The predicted mean cost for the group with clinically significant pain was higher than of the predicted mean cost for the NP/NDPM group. Although differences in sample population, time period, and costing methodologies make it difficult to compare with other studies, collectively our findings are in line with the literature showing that direct medical care costs are higher among patients with pain than patients without pain [12–14]. With an aging population and increasing prevalence of chronic diseases in Canada, we expect these costs to rise in the future.

Although the mean differences in cost as a function of pain status are statistically significant, our estimate of the cost of pain for older adult LTC residents is conservative for several reasons. First, our estimates only captured the direct medical care cost and did not measure the indirect cost of pain such as emotional consequences and suffering which can lower a person’s quality of life. Second, the calculation of LTC cost does not consider the intensity of services required for a given resident. Third, the prescription drug database did not capture information about non-prescription drug costs.

Our pain estimates may be underestimated since the pain items of the RAI-MDS rely primarily on front line staff subjective opinion rather than systematic measurement of pain behaviors. This is particularly important in our study where we are focusing on LTC residents, some of whom are unable to report on their own pain experience due to cognitive impairment. Our results for CPS suggest that residents with severe cognitive impairment had a lower average cost per day than those whose CPS score indicate less impairment. One plausible explanation for such results could be that those with severe impairment are less likely to report pain and other symptoms due to limited communication abilities. Consequently, pain and other symptoms are less likely to be detected/recognized and, therefore, less likely to be treated, resulting in lower care costs. While we found this to be true over the one-year span of our study, it is possible that, over the longer term, undertreated pain could result in higher health care costs as health problems, with potential to increase in severity in the longer term, could go undetected in the short-term (leading to lower short term costs).

The existing literature suggests that pain in people with moderate to severe dementia can be underestimated by front line staff [46]. It is plausible that there may have been a significant portion of CSP residents who were misclassified based on the RAI-MDS. Future research should evaluate cost estimates for pain residents with severe dementia on the basis of systematic pain assessments that include well validated observational tools [47]. Moreover, the literature has documented a frequent undertreatment of pain in people with dementia and this undertreatment would have resulted in reduced health care costs. For example, several studies have indicated that patients with dementia are less likely to receive analgesics than their cognitively intact counterparts [48–50], although this may vary as a function of setting. Attitudinal barriers to effective pain assessment and management in dementia continue to exist [51].

In summary, pain in LTC increases health care costs substantially. Improved pain assessment and management have the potential of reducing such costs and improving life quality for residents. We also believe that the identified cost difference between residents with CSP and those with NP/NDMP is likely underestimated due to the strong possibility that pain problems are often missed among many residents with severe dementia and limited ability to communicate verbally. Future longitudinal clinical research, involving frequent and systematic pain assessments of residents, could potentially provide more accurate estimates of cost difference than we were able to obtain from administrative databases.

### Study limitations

In this study, we classified residents based on pain status as determined by the RAI-MDS pain scale. Although this scale has been validated against resident self-report [34], conclusions are based on nurse opinion and may not be as valid for residents who cannot self-report pain due to aphasia or severe cognitive impairment. Moreover, given that the scale relies heavily on clinician opinion rather than objective pain indicators, we did not feel that it has sufficient sensitivity to identify fine gradations in pain experience. As a result we used an approach that classified the patients into two pain status groups (CSP and NP/NDMP). As such, our analysis does not allow us to evaluate whether there is a direct dose-response relationship between amount of pain experienced and costs. This may be possible to determine in future research that involves other types of pain indices that may be able to reflect accurately gradations in the pain experience [52].

Finally, some variables that may be relevant to cost determination (e.g., facility size, owner/operator model) were not available in our data set. Given that a large proportion of Saskatchewan residents live in rural areas, our results may not generalize to all LTC populations in Canada. A wide body of evidence [53] suggests significant disparities in the health status and quality of care received between rural and urban populations. Future research should extend our work using national-level data to understand any inter-regional variations in the cost of care for pain residents.

## Conclusion

Clinically significant pain in LTC is associated with greater health care costs. Related to this, research has demonstrated that there are significant knowledge gaps among LTC and other health professional staff when it comes to pain assessment and management [54, 55]. The policy implications of our findings are clear: As has been suggested by others [56], allocation of resources of for pain education and development of mandated minimum standards for improved pain assessment and management in LTC are necessary, not only for the quality of life of residents, but also for potential long-term cost savings. Earlier pain detection and treatment may result in future cost savings. Although in this investigation we were not able to consider the impact of pain treatment on health care spending, as demonstrated, outside LTC environments [57], early and effective identification and treatment of pain may result in substantial cost savings, improved quality of life, and prevention of health deterioration. With the rising cost of health care and limited health care budgets, the impact of such treatments in LTC environments merits further study.

## Supplementary Information

**Additional file 1: Supplementary Table 1**. Baseline pain frequency and intensity stratified by sex.

## Data Availability

The data that support the findings of the study are available from the Health Quality Council of Saskatchewan but restrictions apply to the availability of these data, which were used under contract for the current study, and so are not publically available. Researchers may access the data by forming a partnership with the Saskatchewan Health Quality Council as indicated on their website: https://www.hqc.sk.ca/research-partnerships/other-research-partnerships.

## Abbreviations

CANSIM: Canadian Socio-Economic Information Management System
CMG: Case-Mix Groups
CCI: Charlson comorbidity index
CSP: Clinically significant pain
CIs: Confidence intervals
CPI: Consumer Price Index
DAD: Discharge Abstract Database
DIN: Drug identification number
GEEs: Generalized estimating equations
GLMs: Generalized linear models
ICD–9: International Classification of Diseases, 9th revision, Canadian enhancement codes
ICD-10-CA: International Classification of Diseases, 10th revision, Canadian enhancement codes
ISCH: Institutional Supportive Care Home
IQR: Interquartile range
LTC: Long-term care
NP: No pain
NDMP: Non-daily mild pain
PHRS: Person Health Registry System
RAI-MDS: Resident Assessment Instrument-Minimum Data Set
RIW: Resource Intensity Weight
SCHS: Special Care Home System
SD: Standard deviations
