# Supplementary material for: Pain-related health care costs for long-term care residents
Source: BMC Geriatr. 2021 Oct 14;21:552. doi: 10.1186/s12877-021-02424-2 (PMC8515764; doi:10.1186/s12877-021-02424-2)
Supplement: Supplementary file 1 — Additional file 1: Supplementary Table 1. Baseline pain frequency and intensity stratified by sex. [file 12877_2021_2424_MOESM1_ESM.docx]

| **Supplementary Table 1 : Baseline pain frequency and intensity stratified by sex** | | | | | | | | | | | | |
| --- | --- | --- | --- | --- | --- | --- | --- | --- | --- | --- | --- | --- |
|  | | Intensity of pain | | | | | | | | | | |
|  |  | No pain | | Mild pain | | Moderate pain | | Excruciating pain | | Row Totals | |  |
|  |  | n (%) | | n (%) | | n (%) | | n (%) | | n (%) | |  |
|  |  | Male | Female | Male | Female | Male | Female | Male | Female | Male | Female |  |
| Frequency  of pain | No pain | 3959(36.3%) | 6939(63.7%) | -- | --- | -- | -- | -- | -- | 3959(36.3%) | 6939(63.7%) |  |
|  | Pain less than daily | -- | -- | 14(35.9%) | 25(64.1%) | 404(29.9%) | 946(70.1%) | 15(32.6%) | 31(67.4%) | 433(30.2%) | 1002(69.8%) |  |
|  | Pain daily | -- | --- | 305(31.4%) | 668(68.7%) | 756(27.8%) | 1960(72.2%) | 142(25.4%) | 417(74.6%) | 1203(28.3%) | 3045(71.7%) |  |
|  | Column totals | 3959(36.3%) | 6939(63.7%) | 319(31.5%) | 693(68.5%) | 1160(28.5%) | 2906(71.5%) | 157(25.9%) | 448(74.1%) | 5595(33.7%) | 10986(66.3%) |  |
